# Pathologic Findings of Cranial Abscesses Involving the Pituitary Gland in Free-Ranging White-Tailed Deer (*Odocoileus virginianus*) in Pennsylvania

**DOI:** 10.3390/ani15030409

**Published:** 2025-02-02

**Authors:** Jack Timmons, Matthew Shaub, Luke Scherer, Ian Gereg, Lauren Maxwell, Lane Potts, Madison Stevens, Madeline Vile, Erica A. Miller, Kevin D. Niedringhaus

**Affiliations:** 1 College of Veterinary Medicine, Cornell University, Ithaca, NY 14853, USA; jrt257@cornell.edu; 2 Wildlife Futures Program, Department of Pathobiology, School of Veterinary Medicine, University of Pennsylvania, Kennett Square, PA 19348, USA; mshaub@vt.edu (M.S.); schererl@vet.upenn.edu (L.S.); ian.gereg@gmail.com (I.G.); lmaxwel@vet.upenn.edu (L.M.); lpotts@upenn.edu (L.P.); stemad@vet.upenn.edu (M.S.); mvile@vet.upenn.edu (M.V.); millerer@upenn.edu (E.A.M.)

**Keywords:** abscess, deer, neurologic disease, *Odocoileus virginianus*, pituitary abscess, *Trueperella pyogenes*, wildlife health

## Abstract

Intracranial abscesses are a common cause of morbidity and mortality in neurologic free-ranging white-tailed deer (*Odocoileus virginianus*). These infections are typically attributed to *Trueperella pyogenes* and occur most often in adult males with concurrent antler and skull infections. A subset of intracranial abscesses in white-tailed deer are localized to the pituitary gland, with previously described reports in five white-tailed deer. In this case series, five additional free-ranging white-tailed deer in Pennsylvania were diagnosed with pituitary abscesses at necropsy. Two cases were female deer, and four cases were yearling or juvenile deer. Two cases presented with conjunctivitis and keratitis due to extension of pituitary abscesses into the retrobulbar and peri-orbital spaces. One case had a concurrent liver abscess. This case series demonstrates a unique clinical presentation of keratitis and conjunctivitis and emphasizes the potentially unique pathogenesis for pituitary abscesses compared to other intracranial abscesses described in white-tailed deer.

## Simple Summary

White-tailed deer (*Odocoileus virginianus*) commonly develop brain abscesses. While most cases occur on the skin, antler, skull, or brain, a subset more specifically affects the pituitary gland. This study describes five cases of pituitary abscesses in free-ranging white-tailed deer, including two with novel ocular manifestations. A review of these cases emphasizes the clinical presentation, gross and microscopic lesions, demographic data, and bacteriology results. Pituitary abscesses may have a unique pathogenesis compared to other intracranial abscesses in deer and should be considered as a source of conjunctivitis and keratitis by diagnosticians, field personnel, hunters, or other outdoor enthusiasts.

## 1. Introduction

Intracranial abscesses are a well-recognized cause of neurologic disease and mortality in white-tailed deer [1,2,3]. The inciting cause has generally been attributed to opportunistic infection by *Trueperella pyogenes*, a commensal bacterium of ruminant skin and mucosal surfaces including the upper respiratory, gastrointestinal, and urogenital tracts [1,4,5]. The classic signalment for this disease is adult male white-tailed deer, with lesion progression thought to be associated with seasonal changes in antler development and associated behaviors such as rubbing and sparring [1,2,3,6]. Aside from antler-associated infections, other speculated sources of infection include subcutaneous abscesses related to tick infestation, otitis, or penetrating injury elsewhere in the body precipitating hematogenous spread [1,2]. Lesions may range from diffuse suppurative meningoencephalitis to discrete abscesses in the brain often focused near the antler pedicles [1,2]. 

A unique subset of intracranial abscesses in deer are localized to the pituitary gland. From 2013–2015, four free-ranging white-tailed deer with neurologic disease in the Southeastern US were diagnosed with pituitary abscesses [7]. Another prior report describes a pituitary abscess in a captive white-tailed deer with chronic selenium toxicosis [8]. Deer with pituitary abscesses from these two previous studies presented with a variety of clinical signs, including lack of fear of humans, hypersalivation, blindness, ataxia, lethargy, recumbency, and poor body condition [7,8]. As with other intracranial abscesses, the exact pathogenesis of pituitary abscesses in deer is not well understood. 

We describe the clinical presentations and pathologic findings of pituitary abscesses in five white-tailed deer from Pennsylvania, USA and expand on the limited published information of this disease in white-tailed deer.

## 2. Case Series Descriptions

Over a two-year period between August 2022 and August 2024, 25 free-ranging white-tailed deer (Odocoileus virginianus) submitted to the Wildlife Futures Program at the University of Pennsylvania were diagnosed with intracranial abscesses. Of these 25 cases, five (20%) had abscesses that were believed to originate from and primarily involve the pituitary gland. These five free-ranging white-tailed deer were found alive and easily approachable with various neurologic signs throughout Pennsylvania, USA, and were subsequently euthanized via gunshot to the chest due to concern for zoonotic neurologic disease. While all carcasses were dissected out and examined in the field, the heads were removed whole and submitted for detailed examinations and additional testing. Body condition was subjectively categorized as ‘poor’, ‘moderate’, or ‘good’ based on prominence of ribs and hip bones as well as extent of internal adipose tissue. Aerobic culture was performed from swabs of suppurative inflammation in 4/5 cases. Anaerobic culture was not performed in any cases. Select cases were also tested for rabies virus by direct fluorescent antibody test of the brainstem and cerebellum, and chronic wasting disease (CWD) was tested by immunohistochemistry according to established and validated protocols at an American Association of Veterinary Laboratory Diagnosticians (AAVLD)-accredited laboratory.

**Figure 1:**
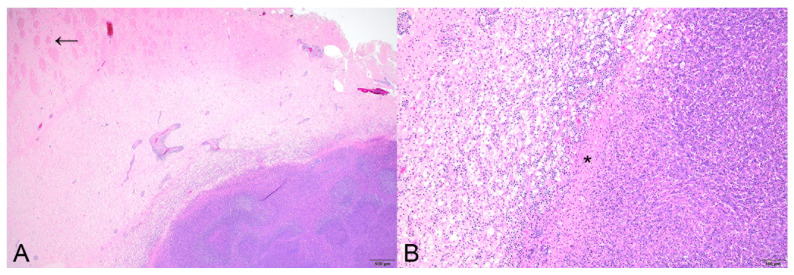
Case 1. Histopathology of a pituitary gland abscess in a white-tailed deer (*Odocoileus virginianus*). (**A**) The abscess extended from the pituitary gland to the hypothalamus; hypothalamic white matter tracts are highlighted by the arrow. H&E; bar = 500 µm. (**B**) Higher magnification of the abscess showing the fibrous capsule (asterisk) separating the abscess from compressed neuropil. H&E; bar = 100 µm.

Case 1. A yearling female white-tailed deer was reported standing and shaking in a yard with closed eyes before lying down prior to euthanasia in Blair County in December 2022. On field necropsy, the deer was in good body condition. Internal examination of the skull revealed a ruptured pituitary abscess within the sella turcica suspected to be ~0.75 cm in diameter with a thin capsule and composed of thick, yellow-green exudate. The regional meninges were dark red. On histopathology, abscessation of the pituitary gland and meninges was characterized by neutrophilic inflammation and abundant nuclear debris extending dorsally towards the hypothalamus (Figure 1A). Surrounding the abscess was a thin fibrous capsule, and the adjacent neuropil was compressed, containing numerous necrotic neurons (Figure 1B). Short Gram-negative rods and nematode larvae (presumed *Parelaphostrongylus tenuis*) were present in inflamed regions. Histopathology also revealed a focal liver abscess like that seen in the pituitary gland, which also contained small clusters of short Gram-negative rods. Portal lymphocytic aggregates, extramedullary hematopoiesis, and dissecting fibrosis were common and most pronounced in regions closest to the abscess. Bacterial culture isolated a moderate amount of mixed organisms including *Escherichia coli*, *Pantoea agglomerans*, *Acinetobacter tandoii*, *Pseudomonas* sp., *Serratia liquefaciens*, and *Carnobacterium* sp. Based on bacterial histomorphology, the interpretation was primarily *E. coli* infection with postmortem overgrowth and/or contamination. Rabies virus and the prion that causes CWD were not detected.

**Figure 2:**
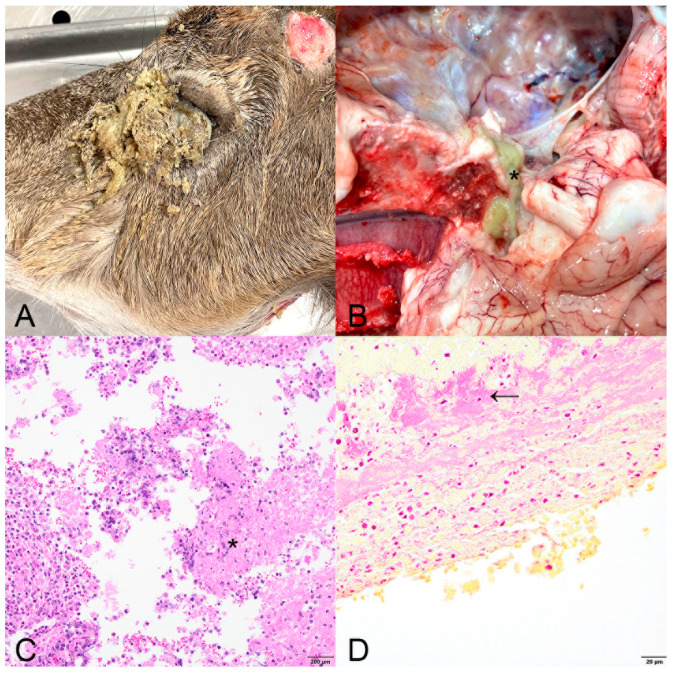
Case 2. Head of white-tailed deer (*Odocoileus virginianus*) showing (**A**) the left eye crusted with severe mucopurulent exudate. (**B**) The same deer with the brain ventrally reflected, revealing yellow-green purulent exudate replacing the pituitary gland (asterisk). (**C**) Inflammation is primarily neutrophilic with streaming nuclear debris and fibrin (asterisk). H&E; bar = 200 µm (**D**). Gram stain highlighting Gram-negative rods (arrow) in one case. Gram stain with McDonald’s modification; bar = 20 µm.

Case 2. A yearling male white-tailed deer was reported walking in circles near a road in January 2023 in Indiana County. The deer had recently shed the right antler, the left antler was hanging low on the head, and the left eye was crusted with dried, yellow exudate. On field necropsy, the deer was in good body condition. The left antler was easily removed during examination. The left globe was coated in thick, yellow, dried exudate that filled the ventral conjunctiva and extended rostrally on the skin (Figure 2A). Internally, the pituitary gland was full of thick, green exudate (Figure 2B). This suppurative inflammation extended into the basisphenoid bone and exited the left orbit and left lacrimal duct where it dried onto the skin. Two red nematodes were within the dura (presumed *P. tenuis*). On histopathology, the abscess was characterized by central necrosis and neutrophilic inflammation of the pituitary gland. Higher magnification identified abundant degenerating neutrophils and streaming nuclear debris (Figure 2C). Short Gram-negative rods were common within the focal abscess with Gram staining (Figure 2D). Elsewhere, neutrophilic exudate expanded and coated the meninges multifocally with moderate meningothelial thickening. Rare blood vessels were characterized by lymphoplasmacytic perivascular cuffing. Histopathology of the eye showed neutrophilic exudate coating and invading through the cornea, resulting in a perforation, and the iris anteriorly displaced and adhered to the posterior surface of the cornea (anterior synechiae). The anterior chamber contained a moderate amount of flocculent eosinophilic debris. The conjunctiva contained abundant lymphocytic inflammation, including tertiary lymphoid follicles, and exhibited moderate epithelial hyperplasia. Neutrophilic inflammation coated the optic nerve focally. Bacterial culture was not performed on this case. Rabies virus and the prion that causes CWD were not detected. 

Case 3. A yearling male white-tailed deer was reported wobbling with blood coming from the left eye in December 2023 in Montgomery County. On field necropsy, the deer was in good body condition. Small purulent foci were present at both antler pedicles that did not communicate with the pituitary gland. Externally, the left eye was protruding from the orbit, and the cornea was cloudy. Internally, the pituitary gland was abscessed with green purulent exudate extending rostrally into the olfactory bulb and left retrobulbar region causing protrusion of the eye. Histopathology was not performed on this case. Bacterial culture grew a moderate amount of *Trueperella pyogenes* and *Staphylococcus aureus*. 

Case 4. An adult female white-tailed deer was reported walking in circles near a road in December 2023 in Susquehanna County. On field necropsy, the deer was in good body condition. The pituitary gland was abscessed with green purulent exudate extending into the adjacent right temporal lobe of the cerebrum. Histopathology revealed lymphocytic and neutrophilic inflammation throughout the meninges and forming small perivascular cuffs in the neuropil. Focal suppurative inflammation was also present in the temporal lobe of the cerebrum. Bacteria were not seen histologically. Moderate growth of *Lelliottia amnigena*, *Aeromonas eucrenophila*, *Escherichia coli*, *Serratia fonticola*, and *Proteus* sp. were isolated by aerobic culture which was interpreted as postmortem overgrowth and/or contamination. Rabies virus and the prion that causes CWD were not detected. 

Case 5. A yearling male white-tailed deer was reported standing inactive in a field in Butler County in January 2024. On field necropsy, the deer was in good body condition, the antlers had been shed, and the pedicles were healed. The pituitary gland was affected by a ~1.5 cm diameter abscess with soft green purulent exudate that extended dorsally into the ventral hypothalamus. On histopathology, the abscess contained degenerate neutrophils and intralesional thin, long, filamentous Gram-negative rods. The lesion was further characterized by central necrosis with compression and necrosis of adjacent neuroparenchyma and neutrophilic perivascular cuffing of surrounding vessels. Aerobic culture isolated light mixed growth, including *Aeromonas hydrophila*, *Pantoea* spp., *Pseudomonas* sp., and *Staphylococcus aureus*. Considering the morphology, the bacteria seen microscopically may be anaerobic and not among the bacteria isolated aerobically. The prion that causes CWD was not detected. 

## 3. Discussion

Pituitary abscesses have been infrequently documented in white-tailed deer. In this study, 20% (5/25) of deer with intracranial abscesses had lesions localized to the pituitary gland. Observed clinical signs in the five affected deer included those reported in other deer and domestic ruminants with pituitary abscesses–lack of fear of humans, ataxia, lethargy, and recumbency [7,9,10]. While all previous reports of deer with pituitary abscesses were in poor bodily condition [7,8], all the deer in our study were in good bodily condition. Additional clinical signs seen in domestic cattle with pituitary abscesses can include dysphagia, ptyalism, and dropped jaw or inability to close the mouth from mandibular paralysis [9,10]. Suspected involvement of the mandibular nerve is attributed to these signs in cattle and could explain the variable wasting seen in some white-tailed deer [10]. 

Gross and histologic characterization of the pituitary abscesses in our study was consistent with previous descriptions of pituitary abscesses in white-tailed deer [7]. In all cases presented here, the pituitary gland was consistently replaced with yellow-green purulent exudate. Determining which portion of the pituitary gland was initially or primarily affected was challenging, largely due to complete effacement or distortion of the gland by suppurative inflammation hindering this interpretation grossly and histologically. In two of the five cases, the abscess extended into the cerebrum through a tract from the pituitary gland. One animal (Case 3) appeared to have separate abscesses at the antler pedicles unrelated to the pituitary gland abscess. Microscopic lesions identified in four of the five pituitary abscesses in this study were characterized predominantly by neutrophilic inflammation with central necrosis. Other prominent histopathologic features included extension of inflammation to the meninges (3/4), compression and malacia of adjacent neuroparenchyma (2/4), formation of perivascular cuffs (3/4), and intralesional Gram-negative rods (3/4). Intracranial nematode larvae were observed in two of the five cases. Morphology and location of nematode larvae were most consistent with *P. tenuis*, though further identification was not performed. This nematode infection is a common finding in white-tailed deer and was reported in another case of pituitary abscesses [7]. The potential role of *P. tenuis* in pituitary inflammation is unclear.

Two of the five deer in this study had extension of pituitary abscesses into the orbit. Of previously reported deer with pituitary abscesses, two cases had bilateral corneal ulceration and retrobulbar abscesses [7]. Another report describes an exudate-filled draining tract behind the left eye of a deer with a pituitary abscess [8]. Unlike previous reports, the two deer in our study presented with ocular manifestations of the abscesses while alive. The clinical manifestations of the orbital abscesses, including crusted yellow exudate and blood coming from the eye, display a unique presentation not previously described in affected deer. Ocular lesions are not typically described in the more common cerebral/antler pedicle intracranial abscesses [1,2]. 

Antemortem ocular signs have been observed in domestic cattle with pituitary abscesses [10,11]. Signs in cattle include unilateral or bilateral exophthalmos, palpebral paralysis, blindness, and ulcerative keratoconjunctivitis; exhibited signs were presumed to be secondary to damage to the oculomotor, trigeminal, and abducent nerves as they exit the cranial cavity. Extension of the pituitary abscess or exudate into the orbit was not observed in calves. The deer in this study had direct extension of abscesses into the peri-orbital region resulting in observed clinical signs. Protrusion of the globe was associated with retrobulbar abscessation, expanding the orbit with purulent exudate. The discharge observed clinically was grossly noted as purulent exudate extending rostrally through the orbit. Given this unique presentation, pituitary abscesses may be considered a differential diagnosis for ocular disease including anterior uveitis, conjunctivitis, and keratitis in white-tailed deer.

Several studies support a significant sex predilection for the development of intracranial abscesses in male deer [1,2,6]. It has been previously speculated that external infection arising from the antlers, incited by shedding, casting, rubbing, or sparring behaviors performed most commonly in males, provides a source for intracranial abscesses. Previous reports of intracranial abscesses in adult male deer describe involvement of the antler pedicles and underlying structures, observed as subcutaneous abscesses and necrosis, erosion, and pitting of cranial bones [2]. In our study, one of the three male deer had mild purulent exudate noted at the antler pedicles on field necropsy. This inflammation was limited to the subcutaneous tissue without clear involvement of bone. Alternative explanation for the pathogenesis of abscess formation is warranted in the absence of lesions in the overlying skin, antler, or skull. Combining this study with both previous reports of pituitary abscesses in deer, a total of six males and four females have been identified. Additionally, five of these ten deer were yearlings or juveniles. This nearly even breakdown of sexes and ages supports a more complex pathogenesis for developing pituitary abscesses compared to other cerebral or skull abscesses previously described. All five cases in this study were documented in December or January, which is consistent with other reports of intracranial abscesses [1,2,6]. Peak rut is mid-November in Pennsylvania [12]; lesions diagnosed in December and January may reflect the time required from infection during the rut to advanced and clinically apparent manifestation several weeks later.

One yearling female (Case 1) in this study had a concurrent liver abscess identified on histopathology, with Gram-negative bacteria with similar morphology detected in both liver and pituitary tissues. Evidence of distant infections amongst the combined 10 reported deer with pituitary abscesses appears to be relatively common, including liver abscesses (2/10), skin lacerations or abscesses (3/10), pulmonary abscesses (2/10), intramuscular abscesses (2/10), salivary gland abscess (1/10), and other visceral abscesses (1/10) [7,8]. Findings of concurrent visceral or cutaneous abscesses may further support a source of hematogenous spread of bacteria to the pituitary gland compared to local invasion suspected in other intracranial abscesses. 

In domestic calves, placement of controlled suckling devices in the nasal septum and subsequent rhinitis has been associated with pituitary abscess formation [10]. Similarly, placement of nose rings may also predispose bulls to pituitary abscesses through a similar mechanism [9]. The pituitary gland is highly susceptible to bacterial localization via the rete mirabile, the intimately associated complex network of vasculature surrounding the pituitary gland; infection from the nasal cavity likely occurs via valveless cerebral veins as well as nasal mucosa lymphatics [10,13]. Given the lack of classic antler-associated lesions in deer in this study and similar findings reported previously, hematogenous spread of opportunistic bacterial infections to the pituitary gland is also a possible route of abscess formation in white-tailed deer. 

Aerobic bacterial culture of pituitary abscesses in this study yielded a wide diversity of bacteria, most of which were considered contaminants or postmortem overgrowth. *Trueperella pyogenes,* the pathogen most frequently detected in intracranial as well as pulmonary and visceral abscesses in white-tailed deer [14,15], was detected in only one of four cases in this study and two of four in the other [7]. Nonetheless, *T. pyogenes* and *E. coli* (3/8) appear to be the most frequently detected bacteria in white-tailed deer pituitary abscesses [7]. Considering the paucity of *Trueperella pyogenes* detections in pituitary abscesses compared with intracranial abscesses, combined with the different sex and age distribution, infections resulting in pituitary abscesses may occur via a unique process compared to true intracranial abscesses in white-tailed deer. 

## 4. Conclusions

Pituitary abscesses in free-ranging white-tailed deer are an important differential diagnosis for neurologic disease with a unique presentation and pathological findings compared to other intracranial abscesses. The pathogenesis remains uncertain in white-tailed deer but likely involves the systemic spread of an opportunistic bacterial infection via the blood to the pituitary gland from local or distant sites. Pituitary abscesses demonstrate the potential to extend into the orbit and should therefore be considered a differential diagnosis for conjunctivitis and keratitis in white-tailed deer. Postmortem examination of white-tailed deer of any age or sex presenting with neurologic or ocular signs should include a thorough examination of the cranial cavity. Further research is indicated to understand the possible origin of bacteria resulting in pituitary abscesses compared to other intracranial abscesses.

## Data Availability

Data are contained within the article.
